# Levetiracetam-Induced Psychosis in the Setting of Intracranial Cavernomas

**DOI:** 10.1155/2022/9114118

**Published:** 2022-03-21

**Authors:** Daniel Majarwitz, Mariam Dvalishvili, Irene Pastis

**Affiliations:** ^1^Department of Psychiatry and Behavioral Medicine, East Carolina University-Vidant Medical Center, Greenville, NC, USA; ^2^Department of Internal Medicine, East Carolina University-Vidant Medical Center, Greenville, NC, USA

## Abstract

Levetiracetam is a commonly used antiepileptic medication that has been associated with the development of psychosis. Cavernomas are vascular malformations that have been associated with psychosis as well, especially in the setting of hemorrhagic transformation. This case report describes a patient with cavernomas who developed psychotic symptoms after restarting her levetiracetam at a high dose (2000 mg twice a day) without gradual uptitration. Her symptoms improved upon the reduction of the levetiracetam as well as the initiation of paliperidone. This case highlights the importance of considering the biologic and medication-related factors for the development of psychosis, as well as the importance of gradual medication adjustments.

## 1. Introduction

Levetiracetam is a commonly used broad-spectrum antiepileptic drug for treatment of partial and generalized seizure disorders. While the mechanism of action has not yet been fully elucidated, it is thought to uniquely inhibit neurotransmitter release by binding synaptic vesicle protein 2A, affecting presynaptic vesicle exocytosis [1]. Compared to the traditional antiepileptics, levetiracetam is generally considered to have a more benign side effect profile, with somnolence and dizziness being some of the most commonly reported reasons for drug cessation [1, 2]. Behavioral disturbances have also been associated with levetiracetam use. White et al. found about 7% of patients stopped taking levetiracetam due to behavioral disturbances, including suicidal ideation and aggression [3]. Reported risk factors for these behavioral abnormalities include psychiatric history and rapid uptitration of levetiracetam [3]. Psychosis has also been documented as one of the neuropsychiatric side effects of levetiracetam initiation [4–6]. Interestingly, psychosis has been documented during planned uptitration as well as accidental overuse of the medication, even in those patients who had been on it long term [7–9].

Cavernomas, or cavernous malformations of the brain, are vascular malformations encased within an endothelium wall. They can enlarge and potentially hemorrhage [10]. The prevalence of cavernomas is about 0.17-0.9%, and the incidence of cavernous malformations appears to be 0.15-0.56 per 100,000 persons per year [11]. Most lesions are supratentorial, and the majority of patients present in the third to fifth decade with seizure as the primary symptom [8]. The mechanism for inciting epileptiform activity appears multifaceted. A potential hypothesis includes the occurrence of small hemorrhages over time that result in accumulation of hemosiderin, calcification, and reactive gliosis, eventually leading to seizures [11, 12].

Here, we report a unique case of suspected levetiracetam-induced psychosis in a patient with intracranial cavernomas.

## 2. Case Description

The patient is a 22-year-old black female with a past medical history significant only for seizure disorder secondary to cavernomas, who initially presented due to bizarre behavior. The patient on initial exams was disorganized and tangential; she was recalling being molested as a child, expressing paranoia about hospital staff, and believing that her aunt and cousin were posing as staff members in order to keep her in the hospital. She was also experiencing auditory hallucinations. Prior to presentation, she had not slept for three days. She had been seizure-free for seven years on a regimen of levetiracetam 2000 mg twice a day and acetazolamide 500 mg twice a day. However, her family reported that the patient had not been compliant with the levetiracetam and that they witnessed motor signs concerning for seizure activity. Per triage documentation from the patient's aunt, there was concern that the patient restarted taking her levetiracetam three days prior to presentation but was not sure of the dose. However, the patient reported she restarted levetiracetam at high dose (2000 mg twice a day) around 3-4 months prior to presentation without any titration.

The patient had been seeing a therapist for anxiety and depressive symptoms prior to admission. She denied any substance use other than “edibles” and occasional alcohol use. Family history was significant for schizophrenia in a family member on the maternal side. She graduated from college and functioned well socially prior to presentation.

**Figure 1:**
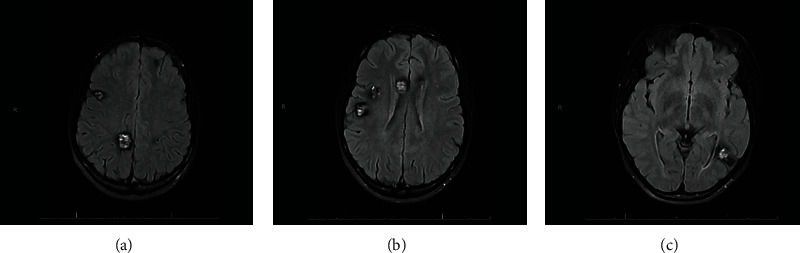
Axial T2 FLAIR sections (superior to inferior) from an MRI performed upon the patient's arrival to the hospital. Various cavernomas are present. The largest lesion in the right cerebral hemisphere is in the paramedian right parietal lobe and measures 15 × 18 mm on axial images and 17 mm superior to inferior (a). The largest lesion is in the posterior left temporal lobe near the temporal occipital junction and measures approximately 19 × 16 mm on axial images and 17 mm superior to inferior (c).

On emergency department evaluation, the physical exam revealed moist mucosa and did not note conjunctival injection. The patient's urine drug screen was positive for cannabis. A continuous electroencephalogram revealed no epileptiform abnormalities. Magnetic resonance imaging (MRI) with and without contrast showed numerous lesions throughout the bilateral cerebral hemispheres, and the brainstem with signal characteristics compatible with multiple cavernomas (see Figure 1). There did not appear to be significant changes since her last MRI three years prior.

The patient required administration of sedative medications multiple times as well as restraints during the initial part of her hospital course due to severe agitation. She continued to show disorganized behavior and was started on 3 mg of paliperidone, which was increased to 6 mg by day four of admission. She was also started on divalproex sodium 500 mg twice a day, and levetiracetam was decreased to 1000 mg twice a day initially. She was continued on acetazolamide 500 mg twice a day. Her psychosis resolved during her hospitalization, and she became more coherent, linear, logical, and insightful. She no longer endorsed delusions or paranoia about family members and was able to participate in group activities. She was discharged on levetiracetam 500 mg twice a day once the valproic acid blood level was found to be in the therapeutic range. She was also discharged on paliperidone 6 mg with the plan to taper off over the next few weeks to months as appropriate. Total length of hospitalization was 13 days.

## 3. Discussion

Here, we describe a case of psychosis in the setting of high-dose levetiracetam use with rapid uptitration in the setting of cavernomas. This case highlights the importance of considering the biologic and medication-related factors for the development of psychosis, as well as the importance of slow titrations when using medications that have psychiatric side-effects. The patient appeared to be intermittently compliant with the levetiracetam and then started retaking the medication at the original, high dose of 2000 mg twice a day. Although the patient had reported restarting levetiracetam a few months ago, her family believed she had only restarted the medication a few days prior to presentation.

An important diagnosis to consider in the differential includes cannabis-induced psychotic disorder, as her urine drug screen was positive for cannabis. Indeed, cannabis is well-associated with psychosis and has been implicated in about 50% of psychosis, schizophrenia, and schizophreniform cases [13]. The main psychoactive component, delta-9-tetrahydrocannabinol (THC), appears to induce positive symptoms of psychosis [14]. Resolution of symptoms occurs without the use of medication when there is abstinence of cannabis [15]. Cannabis intoxication symptoms, which include behavioral and psychological changes in addition to physical signs (i.e., dry mouth and conjunctival injection), usually last about three to four hours but can continue up to 24 hours [16]. Psychotic symptoms can present during this time, but insight is intact, and the psychosis is not severe enough to necessitate clinical evaluation. If the psychotic symptoms are severe enough to lead to medical evaluation, then cannabis-induced psychotic disorder can be considered. In this condition, hallucinations and delusions occur without insight, and the disorder can last for days to weeks after cannabis use [16]. Per the Diagnostic and Statistical Manual of Mental Disorders-V (DSM-V), cannabis-induced psychotic disorder can occur after high-dose drug use and usually resolves within a day but can continue for a few days [17].

Cannabis may have played a role in her symptoms. However, the initial exam did reveal moist oral mucosa and no physical signs of marijuana ingestion. The patient's symptoms started three days prior to her emergency room visit, consistent with the timeline provided by the family, and persisted for days during her hospitalization. Although the effects of the drug use could linger for up to weeks, as mentioned in the DSM-V, the psychotic symptoms usually last for a day to a few days. Additionally, her symptoms improved upon initiation of an antipsychotic and reduction of the levetiracetam dosage. The patient did admit to daily cannabis use at her outpatient follow-up visit after her hospitalization. Psychosis in the setting of cannabis use can recur when ingested again [18], but the patient did not experience any more hallucinations or delusions during her follow-up course. The patient was continued on paliperidone for approximately three months after her discharge and was weaned off without recurrence of psychotic symptoms. Although psychotic features may develop during regular use of cannabis, the recent levetiracetam adjustments most likely provoked the aforementioned psychotic episode, which would explain why the patient presented at the time which she did.

Another possibility to consider is psychosis secondary to mass effect or bleeding from a cavernoma. Case reports have been documented regarding psychosis secondary to cavernomas and tend to be in the setting of hemorrhagic transformations [19, 20]. Although the MRI done upon admission showed stability in the cavernomas and no hemorrhages, the observed vascular malformations may have put her at a risk for psychotic development. Additionally, the presence of microhemorrhages may have caused epileptic activity, resulting in behavioral manifestations. However, the patient did not have any other signs or symptoms consistent with seizure activity, and her electroencephalogram was negative. An additional potential diagnosis includes a brief psychotic disorder and the development of a true thought disorder; however, the patient did not exhibit prodromal symptoms indicative of schizophrenia or related spectrum disorders. Her symptoms appeared suddenly and in the context of restarting levetiracetam.

In a retrospective study by Pinckaers et al., they found that 34.1% of psychotic episodes during treatment for epilepsy were directly attributed to the presence of levetiracetam [21]. Perhaps psychosis secondary to levetiracetam is more common than previously appreciated. While the exact mechanism of levetiracetam is still being studied, its primary and unique mechanism of action appears to be related to binding the synaptic vesicle protein, resulting in attenuated neurotransmitter release [22]. Conceivably, the sudden change in the concentration of neurotransmitter release could result in psychiatric manifestations, such as aggression, suicidal behavior, or psychosis. Future studies should help elucidate the connection between neurotransmission concentrations and behavioral disturbances.

## 4. Conclusion

This case report demonstrates an association between high-dose levetiracetam without gradual titration and the development of psychotic features in a patient with intracranial cavernomas. Physicians should always consider the biologic and medication-related risk factors that may contribute to the development of psychosis, as well as the importance of gradual titrations when using medications with potential psychiatric side-effects.

## Data Availability

The patient data used to support the findings of this study have not been made available because of the need for patient confidentiality. All clinical information is located on the patient's electronic medical record.

## References

[1] Lyseng-Williamson K. A. (2011). Levetiracetam: a review of its use in epilepsy. *Drugs*.

[2] Abou-Khalil B. (2008). Levetiracetam in the treatment of epilepsy. *Neuropsychiatric Disease and Treatment*.

[3] White J. R., Walczak T. S., Leppik I. E. (2003). Discontinuation of levetiracetam because of behavioral side effects: a case-control study. *Neurology*.

[4] Zaki S. A., Gupta S. (2014). Levetiracetam-induced acute psychosis in a child.. *Indian journal of pharmacology*.

[5] Kossoff E. H., Bergey G. K., Freeman J. M., Vining E. P. G. (2001). Levetiracetam psychosis in children with epilepsy. *Epilepsia*.

[6] Erdogan S., Bosnak M. (2017). Hallucination: a rare complication of levetiracetam therapy. *Northern clinics of Istanbul*.

[7] Ogunsakin O., Tumenta T., Louis-Jean S. (2020). Levetiracetam induced behavioral abnormalities in a patient with seizure disorder: a diagnostic challenge. *Case Reports in Psychiatry*.

[8] Dannaram S., Borra D., Pulluri M., Jindal P., Sharma A. (2012). Levetiracetam-induced acute psychotic episode. *Innovations in clinical neuroscience*.

[9] Bayerlein K., Frieling H., Beyer B., Kornhuber J., Bleich S. (2004). Drug-induced psychosis after long-term treatment with levetiracetam. *The Canadian Journal of Psychiatry*.

[10] Rosenow F., Alonso‐Vanegas M. A., Baumgartner C. (2013). Cavernoma-related epilepsy: review and recommendations for management—report of the surgical task force of the ILAE commission on therapeutic strategies.. *Epilepsia*.

[11] Goldstein H. E., Solomon R. A. (2017). Epidemiology of cavernous malformations. *Handbook of Clinical Neurology*.

[12] Kim D.-S., Park Y.-G., Choi J.-U., Chung S.-S., Lee K.-C. (1997). An analysis of the natural history of cavernous malformations. *Surgical Neurology*.

[13] Shrivastava A., Johnston M., Terpstra K., Bureau Y. (2014). Cannabis and psychosis: neurobiology. *Indian Journal of Psychiatry*.

[14] D'Souza D. C., Perry E., MacDougall L. (2004). The psychotomimetic effects of intravenous delta-9-tetrahydrocannabinol in healthy individuals: implications for psychosis. *Neuropsychopharmacology*.

[15] Kulhalli V., Isaac M., Murthy P. (2007). Cannabis-related psychosis: presentation and effect of abstinence. *Indian Journal of Psychiatry*.

[16] Pearson N. T., Berry J. H. (2019). Cannabis and psychosis through the lens of DSM-5. *International Journal of Environmental Research and Public Health*.

[17] American Psychiatric Association (2013). *Diagnostic and Statistical Manual of Mental Disorders*.

[18] D'Souza D. C. (2007). Cannabinoids and psychosis. *International Review of Neurobiology*.

[19] Sayadnasiri M., Fadai F. (2016). Multiple cavernous angiomas associated with psychotic symptoms: a case report. *Zahedan Journal of Research in Medical Sciences*.

[20] Pavesi G., Causin F., Feletti A. (2014). Cavernous angioma of the corpus callosum presenting with acute psychosis. *Behavioural Neurology*.

[21] Pinckaers F. M., Boon M. E., Majoie M. H. (2019). Risk factors predisposing to psychotic symptoms during levetiracetam therapy: A retrospective study. *Epilepsy & Behavior*.

[22] Yang X.-F., Weisenfeld A., Rothman S. M. (2007). Prolonged exposure to levetiracetam reveals a presynaptic effect on neurotransmission.. *Epilepsia*.

